# Effects of *Valeriana officinalis* (Valerian) on tension-type headache: A randomized, placebo-controlled, double-blind clinical trial 

**Published:** 2020

**Authors:** Hossein Azizi, Asie Shojaii, Fataneh Hashem-Dabaghian, Mohammadreza Noras, Amirreza Boroumand, Bita Ebadolahzadeh Haghani, Roshanak Ghods

**Affiliations:** 1 *Research Institute for Islamic and Complementary Medicine, School of Persian Medicine, Iran University of Medical Sciences, Tehran, Iran*; 2 *Faculty of Persian and Complementary Medicine, Mashhad University of Medical Sciences, Mashhad, Iran*; 3 *Department of Neurology, Shams Hospital, Mashhad, Iran*; 4 *Faculty of Persian and Complementary Medicine, Mashhad University of Medical Sciences, Mashhad, Iran*

**Keywords:** Tension-type headache, Valeriana officinalis, Persian medicine, Clinical trial

## Abstract

**Objective:**

Tension-type headache is the most frequent type of headache. Considering the effectiveness of *Valeriana officinalis *(Valerian) in treatment of some types of headache, the effect of valerian root was studied in patients with tension-type headache.

**Materials and Methods:**

The current study is a double-blind randomized clinical trial that was conducted in Shams Hospital of Mashhad University of Medical Sciences, Mashhad, Iran, from January to June 2018. We included 88 participants with tension-type headache and randomly assigned them to intervention and control group by block randomization in a 1:1 ratio. The intervention group received Sedamin® capsule (530 mg of valerian root extraction) while the placebo group received 500 mg of breadcrumbs both given as two capsules daily for a month -after dinner. The headache impact on activity of daily livings performance, headache disability, and headache severity were measured using questionnaires in baseline and one month after intervention in both groups.

**Results:**

The average age (±SD) of the participants was 34.9 (±8.7) years old. After one month, the impact of headache on daily livings performance, significantly reduced in intervention group (mean=51.2) versus the placebo (mean=57.0), (p<0.001). There was a significant reduction in disability in intervention group (mean=22.9) compared to the placebo (mean=27.4) (p<0.001) and the severity score showed significant reductions in intervention group (mean=3.5) versus the placebo group (mean=5.1) (p<0.001).

**Conclusion:**

The present trial showed that valerian capsule could reduce the headache impact on daily livings performance, disability and severity of tension-type headache.

## Introduction

Headache is one of the most common medical complaints and disorders, worldwide (Kasper et al., 2016 ▶). Headache disorders, as major health problems, impose considerable social, emotional and economic burden on sufferers due to life impairment and disabilities which consequently involve high costs in healthcare systems (Balaban et al., 2012 ▶; Nazari and Mahmudi, 2010 ▶). Tension-type headache is known as the most common type of headache accounting for 69% of all cases (Kasper et al., 2016 ▶). Furthermore, the underlying etiology of tension-type headache is poorly understood and chemical therapies for this type of headache are usually palliative and symptomatic. Nevertheless, there are some studies showing the benefits of medicinal herbs on the treatment of various types of headache (Ghorbanifar et al., 2014 ▶). 

According to the WHO, “*Traditional medicine is used in the maintenance of health as well as in the prevention, diagnosis, improvement or treatment of physical and mental illness*” (WHO, 2000). Persian medicine is a kind of traditional medicine and its most important approach is finding the fundamental causes of diseases on one side and studying the best treatment strategy on the other side. For example, in Persian medicine, impairment of the gastric function and lower esophageal sphincter (LES), and brain dysfunction, are among the important causes of headache. It seems that plants that strengthen the LES and improve stomach function, as well as the brain tonics can play a role in reducing headache. Since valerian can strengthen the LES, it can be a good choice for the treatment of headache because of its tonic effects on gastric function and LES.

*Valeriana officinalis* L. (Valerian) is a plant from the Caprifoliaceae family, which is native to different temperate climates of Europe, Asia and America (Isetts, 2007 ▶). Recent studies assessed the effects of valerian on different health outcomes (**Nandhini et al., 2018**; Isetts, 2007 ▶). So far, in herbal medicine, the roots of *V. officinalis* have been utilized as a seductive, antispasmodic agent (Oshima and Ohizumi, 1995 ▶), anxiolytic, and antidepressant and against cardiac arrhythmia (Jia, 1999) and sleep disorders (Becker, 1985 ▶). The previous clinical trials reported conclusive findings about the effect of valerian on the migraine headache (Mirzaee et al., 2015 ▶), but the effect of valerian on the tension-type headache still is not clear. 

Based on the evidence on the effectiveness of valerian in migraine headache and since there is no reports about direct relationship between valerian and tension-type headache, therefore, in the present study, the effectiveness of valerian on tension-type headache was evaluated in a double-blind randomized placebo-controlled trial.

## Materials and Methods

**Trial design**

The present study was designed as a double-blind placebo-controlled randomized clinical trial (RCT) that was done in accordance with the 2010 Consort Guidelines and conducted in the neurology clinic of Shams Hospital of Mashhad University of Medical Sciences, Mashhad, Iran, from January to June 2018. This clinical trial was approved by the ethics committee of Iran University of Medical Sciences (IUMS), Tehran, Iran (No. IR.IUMS.FMD.REC1396.9321309012) and registered at Iranian Registry of Clinical Trials (IRCT) under the registration code IRCT20171203037738N1. We included 88 patients suffering from tension-headache in this RCT. The patients were randomly allocated in two groups equally using the blocked randomization (four blocks) method. The numbers were written on cards inside sealed, opaque envelopes and were kept by a secretary who was not aware of A&B codes and A&B drug box.

First, all patients who referred to the clinic were evaluated to select those who meet the inclusion criteria. Second, after clinical evaluations done by a neurologist and checking the inclusion criteria, they were introduced to a trained secretary. Next, the secretary allocated a random card A or B to them. In the final stage, all the included participants were referred to the investigator and they filled a self-administered questionnaire and received intervention based on the card received. In addition, considering the double-blind nature of the study, neither the researcher nor the patients knew which card they had got. All participants signed a written informed consent. The duration of the intervention was one month and researcher was on call (24 hr) during the whole study period. Compliance with treatment during the study was assessed and further reinforced by the investigator in weekly direct contact with all participants. Patients were visited weekly by a neurologist during the study period. If a patient felt a need to take a daily painkiller due to severe headache, then, that participant would be excluded during the intervention. After one month, both groups were invited to the clinic and were asked to fill the same questionnaire again. During the study, participants continued their previous medications.

**Drug and placebo preparation **

The dried roots of *V.* *officinalis* known as medicinal part of plant were considered in this study (Isetts, 2007 ▶). Capsules containing *V.* *officinalis* root (Sedamin capsule) were purchased from Goldaru pharmaceutical company, Isfahan, Iran. Each capsule contained 530 mg of valerian root extraction. Its health code was 1228022753 and medication barcode was 6260232370041. The valerian and placebo capsules were similar in size, appearance, and color and marked with code A and B. Sixty capsules were given to the participants (to take two capsules daily after dinner) for one month.

**Participants**

All the patients with headache who came to the clinic, were evaluated by the neurologist on the basis of the International Classification of Headache Disorders criteria (ICHD-III) (Olesen, 2018 ▶). The inclusion criteria were being 18 to 60 years old and suffering from frequent episodic or chronic tension-type headache based on ICHD-III. 

Exclusion criteria were as follows: participants with uncommon or probable tension-type headache; other types of primary or secondary headaches; history of taking tryptamine-based/ barbiturates/sedative drugs; pregnancy/lactation; history of chronic diseases including diabetes, hypertension, or heart failure; patients who were using prophylactic medications; patients who had to use daily pain killers during the study period. The participants who met the eligibility criteria, were referred to the study secretary, and according to the previously determined randomization list, randomly allocated to the envelopes containing A or B codes. 

**The primary and secondary endpoints**

Demographic data such as age, gender, weight, height, marital status, job title, educational level, and medical and drug history were collected and recorded during the first interview with the participants.

The headache impact on performance of daily livings 

was the primary endpoint which was measured using headache impact test questionnaire (HIT-6) (Bjorner et al,. 2003 ▶; Nachit-Ouinekh et al., 2005 ▶). This questionnaire consists of six questions designed by Kosinski (Kosinski et al., 2003 ▶), and its Persian version was translated and validated by Zandifar et al. (Zandifar et al., 2014 ▶) with a Cronbach's alpha coefficient of 0.74. The secondary outcome was the headache disability inventory (HDI) (Jacobson et al., 1994 ▶) that was measured by HDI questionnaire. This questionnaire includes 25 questions )13 emotional and 12 functional factor questions), and was designed by Jacobson et al. (Jacobson et al., 1995 ▶); it was translated to Persian by Sajadinezhad et al. (Sajadinezhad et al., 2007 ▶) with a Cronbach's alpha coefficient of 0.77. In addition, the severity of headache was measured using Visual Analogue Scale (VAS) ranging from 0 to 10 (Carlsson, 1983 ▶). All the three questionnaires were completed by the participants of both intervention and control groups before and after the intervention (with one-month interval).

**Sample size**

Based on previously studies, the sample size was calculated by a statistician considering 0.80, 0.05, 0.6 and 20% as power, alpha, effect size and attrition rate, respectively and 44 patients were allocated to each of the intervention and control group.

**Statistical methods**

Data normality was assessed using the Kolmogorov-Smirnov test. As an intent-to-treat (ITT) analysis, Mann–Whitney U test, independent samples t-tests, paired t-test, Wilcox, and chi-square tests were used for bivariate comparisons between groups. Statistical analysis was done using SPSS 16 software (SPSS for Windows, Version 16.0. Chicago, SPSS Inc., 2007). 

## Results

**Baseline measurements**

**Figure 1:**
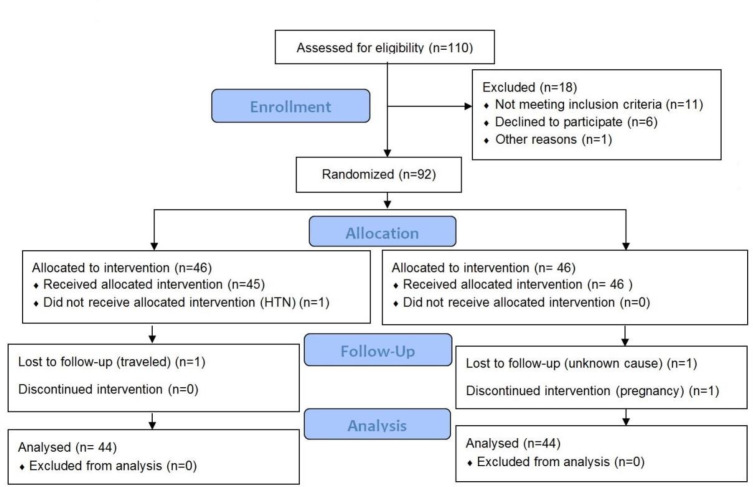
The flowchart of the randomized clinical trial for assessing the effect of valerian on headache reduction

From January to June 2018, 110 patients were recruited for the present trial. Out of 110 participants, 22 did not satisfy the inclusion criteria and were therefore excluded from the study. The reasons for exclusion were: not eligible based on inclusion criteria (n=11), declined to participate (n=6), loss to follow-up (n=3), history of hypertension (n=1) and pregnancy (n=1). Finally, 88 persons were included in the study and randomly allocated to treatment (n=44 participants) and control group (n=44) (Figure 1). The mean age of the participants was 34.9±8.7 years old (mean±SD). No significant differences were detected in demographic data between two groups regarding (p>0.05) (Table 1).

**Table 1:** Demographics characteristics of the participants in treatment and control groups

**Variables **		**Study groups**		**P-value**
		**Valerian**	**Placebo**	
**Age (year)**		33.5±8.5	36.34±8.78	0.13
**Sex**	Male	6 (13.6)	10 (22.7)	0.26
	Female	38 (86.4)	34 (77.3)	
**Education**	≤Diploma	30 (68.2)	36 (81.8)	0.14
	>Diploma	14 (31.8)	8 (18.2)	

**Main findings**

**Figure 2:**
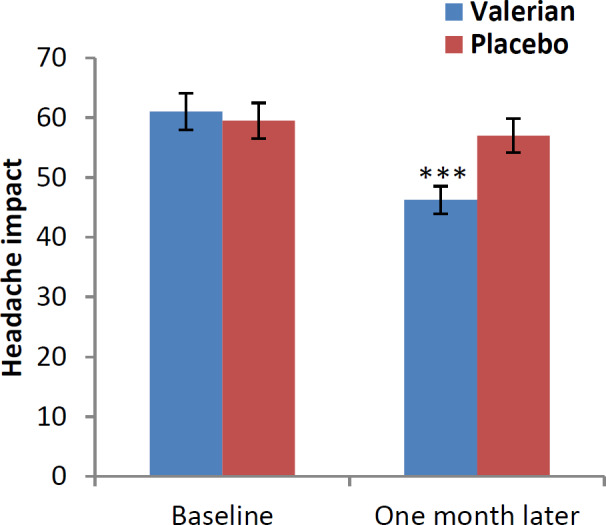
Impact of headache in both groups at baseline and one month after (Headache Impact Test (HIT-6)). ***p<0.001, show significant difference between baseline values and one month after intervention

**Figure 3:**
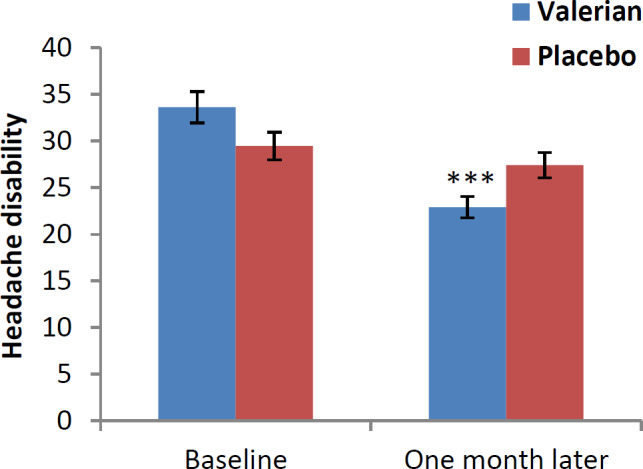
Disability of headache in both groups at baseline and one month after (Headache Disability Inventory (HDI)). ***p<0.001, show significant difference between baseline values and one month after intervention

**Figure 4:**
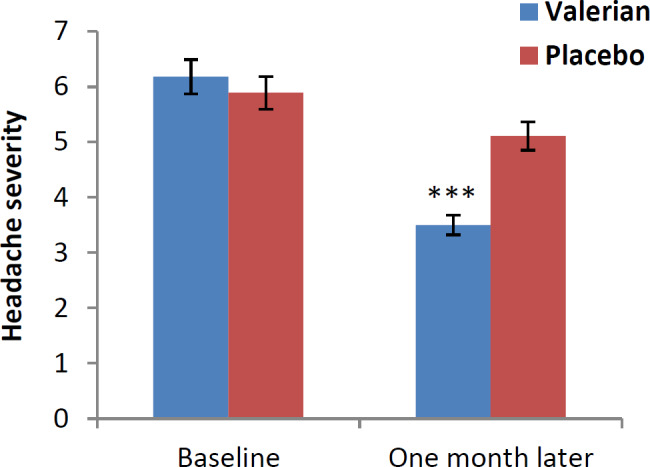
Severity of headache in both groups at baseline and one month after (visual Analogue Scale (VAS)).***p<0.001, show significant difference between baseline values and one month after intervention

The mean difference and 95% confidence interval (CI 95%) of HIT-6, HDI and VAS questionnaires in baseline and one month after intervention are shown in Figures 2, 3 and 4. After one month of treatment, headache impact on performance of daily livings, measured using HIT-6, was significantly lower in intervention compared to control groups (p<0.001) (Figure 2). In addition, we found similar significant reduction in disability scale in intervention versus the control group (p<0.001) (Figure 3). Likewise, significant reduction was found in severity scale in intervention compared to control group (p<0.001) (Figure 4).

**Adverse events**

No drug complications were reported in the treatment and control groups during the study period. However, one participant in valerian group was diagnosed with dizziness at the beginning of the treatment. Therefore, we divided the dosage of intervention to one capsule at night and another in the morning.

## Discussion

In this study, the effect of valerian on tension-type headache was evaluated and compared with placebo in a randomized, double-blind, placebo-controlled study. Findings showed that valerian capsule has a significant effect on the severity and disability of tension-type headaches compared to placebo. This finding was obtained by three HIT-6, HDI, and VAS questionnaires which measured the endpoints before and after the intervention.

The pharmacological effects of valerian are attributed to the constituents of volatile oils, monoterpenes, valepotriates and sesquiterpenes (valerenic acid) (Nandhini et al., 2018). Some of these constituents were reported to act directly on the brain while valerenic acid inhibits enzyme induced breakdown of γ-amino butyric acid (GABA) in the brain which finally results in sedation (Isetts, 2007 ▶). Therefore, valerian consumption can lead to inhibiting the reabsorption of GABA in the brain and consequently sedation and can have a considerable effect on reducing the daily stress. Considering the fact that one of the most important risk factors for tension-type headache is stress, one possible justification for valerian effect on tension-type headache could be reducing stress pathway. Therefore, drugs that have positive effects on stress, can play a role in improving tension-type headaches.

According to Persian medicine, valerian plays a role in relieving headaches by improving the brain function. Several studies showed that valerian exerts a significant positive effect on treatment of problems such as insomnia, bad sleep, anxiety, and depression. There are some studies about the analgesic effect of valerian on migraine and dysmenorrhea. In a randomized clinical trial study, the effect of *V. officinalis *capsules in participants with migraine attacks who were previously treated with sodium valproate was evaluated; the results indicated that valerian capsule significantly reduced the frequency, duration, and intensity of migraine attacks which is consistent with the results of this study. In another study, the effect of complementary plant supplements (Indian valerian and *Melissa officinalis*) on migraine was evaluated for eight weeks. The finding showed that in this group, eight weeks of activity significantly reduced the number of headache days, headache severity, anxiety and depression symptoms which refer to valerian's effect on the migraine headache. (Mirzaee et al., 2015 ▶). Taavoni et al. found that Sedamin capsule (valerian) can improve the quality of sleep in menopausal women with the sleep disorder (Taavoni et al., 2011 ▶). In the present study, one-month consumption of valerian had a significant effect on tension-type headache which was comparable to the analgesic effect of this plant in migraine attack and dysmenorrhea (Taavoni et al., 2011 ▶).

Similar studies in children and adolescents with different doses of valerian could be designed. Phytochemical study for indicating the active compounds of valerian in tension-type headache is recommended. 

In the present study, we showed that valerian capsule could reduce the headache impact on performance of daily livings, severity, and disability of a tension-type headache. So, it can be an effective treatment that could be recommended as a therapy to the patients who suffer from this type of headache along with other common drugs.
